# Crystal structures of adenylylated and unadenylylated P_II_ protein GlnK from *Corynebacterium glutamicum*

**DOI:** 10.1107/S2059798321000735

**Published:** 2021-02-19

**Authors:** Florian C. Grau, Andreas Burkovski, Yves A. Muller

**Affiliations:** a Division of Biotechnology, Department of Biology, Friedrich-Alexander-Universität Erlangen-Nürnberg, Henkestrasse 91, 91052 Erlangen, Germany; b Division of Microbiology, Department of Biology, Friedrich-Alexander-Universität Erlangen-Nürnberg, Staudtstrasse 5, 91058 Erlangen, Germany

**Keywords:** nitrogen starvation, bacterial signal transduction, post-translational modifications, AMPylation, adenylylation, crystal structures, T-loop conformations

## Abstract

P_II_ proteins are ubiquitous signaling proteins that are involved in the regulation of the nitrogen/carbon balance in bacteria, archaea, and some plants and algae. Signal transduction *via* P_II_ proteins is modulated by effector molecules and post-translational modifications in the P_II_ T-loop. Whereas the binding of ADP, ATP and the concomitant binding of ATP and 2-oxoglutarate (2OG) engender two distinct conformations of the T-loop that either favor or disfavor the interaction with partner proteins, the structural consequences of post-translational modifications such as phosphorylation, uridylylation and adenylylation are far less well understood. In the present study, crystal structures of the P_II_ protein GlnK from *Corynebacterium glutamicum* have been determined, namely of adenylylated GlnK (adGlnK) and unmodified unadenylylated GlnK (unGlnK). AdGlnK has been proposed to act as an inducer of the transcription repressor AmtR, and the adenylylation of Tyr51 in GlnK has been proposed to be a prerequisite for this function. The structures of unGlnK and adGlnK allow the first atomic insights into the structural implications of the covalent attachment of an AMP moiety to the T-loop. The overall GlnK fold remains unaltered upon adenylylation, and T-loop adenylylation does not appear to interfere with the formation of the two major functionally important T-loop conformations, namely the extended T-loop in the canonical ADP-bound state and the compacted T-loop that is adopted upon the simultaneous binding of Mg-ATP and 2OG. Thus, the P_II_-typical conformational switching mechanism appears to be preserved in GlnK from *C. glutamicum*, while at the same time the functional repertoire becomes expanded through the accommodation of a peculiar post-translational modification.

## Abstract 2

The crystal structures of adenylylated and unadenylylated GlnK, a P_II_ protein from *Corynebacterium glutamicum*, indicate that adenylylation of Tyr51 in the T-loop does not interfere with the P_II_-typical conformational changes that occur in the T-loop upon effector binding. Rather, T-loop adenylylation further expands the repertoire of mechanisms that enable P_II_ function.

## 1. Introduction   

The P_II_ protein family consists of a group of ubiquitous signal transduction proteins that are not only present in proteobacteria, actinobacteria and cyanobacteria, but are also found in archaea and in the chloroplasts of certain algae and plants (Huergo *et al.*, 2003 ▸). P_II_ proteins have not been observed in animals and fungi to date (Merrick, 2014 ▸). Whereas many organisms encode multiple P_II_ paralogues, some organisms, such as most cyanobacteria, harbor only a single P_II_ protein (Merrick, 2014 ▸).

The prototypical P_II_ proteins GlnB and GlnK are among the best-studied P_II_ proteins. They function as sensors of the nitrogen/carbon status and actively participate in the regulation of cellular nitrogen/carbon uptake. Sensing is achieved *via* the interaction of the P_II_ protein with specific effectors such as ADP, Mg-ATP and 2-oxoglutarate (2OG). In addition, post-translational modifications such as uridylylation, adenylylation and phosphorylation may also occur (Merrick, 2014 ▸). The formation of P_II_–effector ligand complexes engenders distinct protein conformational states (Forcada-Nadal *et al.*, 2018 ▸; Forchhammer & Selim, 2020 ▸; Fokina *et al.*, 2010 ▸; Truan *et al.*, 2010 ▸). The ADP-bound state is formed when cellular 2OG concentrations are low. This also signals high availability of nitrogen, since nitrogen abundance causes the conversion of 2OG to l-glutamate and hence the depletion of 2OG (Forchhammer & Selim, 2020 ▸). Conversely, the complex with Mg-ATP and 2OG is formed when 2OG concentrations are high, in testimony of an abundance of carbon and a shortage of nitrogen. These distinct states act as a nitrogen/carbon balance controller through the formation of effector-specific protein–protein complexes and the concomitant regulation of the activity of transporters, enzymes and transcription factors (Forcada-Nadal *et al.*, 2018 ▸; Forchhammer & Selim, 2020 ▸).

**Figure 1:**
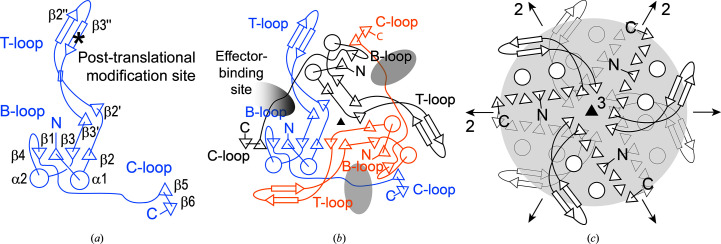
Canonical structure of P_II_ proteins. (*a*) Topology plot of a P_II_ monomer. Structural features such as the B-, C- and T-loops, as well as the post-translational modification site located in the T-loop, are highlighted. (*b*) P_II_ protein trimer displaying *C*_3_ point-group symmetry. Each protomer is depicted in a different color. (*c*) Hexameric assembly with *D*_3_ point-group symmetry of two P_II_ trimers as observed in the crystal structures of unGlnK and adGlnK from *C. glutamicum*. The symmetry elements present in point groups *C*_3_ and *D*_3_ are illustrated as follows: black triangles indicate threefold rotation axes oriented perpendicular to the plane of the illustration and black arrows indicate twofold rotation axes located in the plane of the illustration.

All P_II_ proteins share a high level of structure and sequence similarity. They form homotrimers with *C*_3_ point-group symmetry (Forcada-Nadal *et al.*, 2018 ▸; Forchhammer & Selim, 2020 ▸; Fig. 1 ▸). Each monomer displays a central four-stranded β-sheet that is flanked on either side by additional β-strands contributed by the two additional subunits present in the trimeric assembly. Effector binding occurs at the interface between subunits and involves interactions with three loop regions, called the B-, C- and T-loops (Fig. 1 ▸). The allosteric signal that links effector binding to the regulation of the activity of P_II_-interacting proteins is primarily transduced *via* the T-loop, which adopts different conformations depending on the nature and combination of bound effectors. The T-loop also harbors all presently known post-translational modification sites (Merrick, 2014 ▸). Among the most important functionally mapped T-loop conformations are the extended conformation that is stabilized upon ADP binding, the semi-variable conformation that is induced by Mg-ATP and the compacted conformation that is induced upon the binding of Mg-ATP and 2OG (Truan *et al.*, 2014 ▸). While the former two conformations enable the interaction of P_II_ proteins with distinct target proteins, the latter conformation is generally considered to abrogate any binding interactions (Fokina *et al.*, 2010 ▸; Truan *et al.*, 2010 ▸; Forchhammer & Selim, 2020 ▸). Additional variations in the T-loop conformation and the binding of additional effectors, such as the binding of AMP and ATP without Mg^2+^, may also occur and have been reported for some P_II_ proteins (Forcada-Nadal *et al.*, 2018 ▸). Moreover, in many plants glutamine acts as an additional effector, albeit binding to a different binding site (Forcada-Nadal *et al.*, 2018 ▸).

In the present study, we determined crystal structures of the P_II_ protein GlnK from *Corynebacterium glutamicum* in its post-translationally modified adenylylated (adGlnK) and unmodified unadenylylated (unGlnK) states. The crystal structure of a similarly modified P_II_ protein, namely that of uridylylated GlnB from *Escherichia coli*, has previously been determined. However, the part of the T-loop carrying the post-translational modification was not resolved in this structure (Palanca & Rubio, 2017 ▸). It has been proposed that adGlnK acts as an inducer of the global bacterial transcription regulator AmtR and thereby regulates nitrogen metabolism in *C. glutamicum* (Beckers *et al.*, 2005 ▸; Strösser *et al.*, 2004 ▸). Deletion of the *glnK* gene abolishes the upregulation of AmtR-controlled genes, and the derepression of genes under AmtR only occurs if GlnK is adenylylated at position 51, since exchanging Tyr51 for phenylalanine resulted in an identical phenotype to those observed for *glnK* deletion mutants (Nolden *et al.*, 2001 ▸; Beckers *et al.*, 2005 ▸). Whereas the interaction between AmtR and its target DNA has been well characterized, the details and structural implications of the adenylylation of GlnK as well as the mechanism by which adGlnK derepresses AmtR-controlled gene transcription currently remain largely elusive (Palanca & Rubio, 2016 ▸; Sevvana *et al.*, 2017 ▸).

## 2. Methods   

### 2.1. Expression and purification of unGlnK and adGlnK   

UnGlnK from *C. glutamicum* (UniProt entry Q79VF2; The UniProt Consortium, 2017 ▸) was expressed in *Escherichia coli* BL21(DE3) cells with an N-terminal His tag. The cells were grown in LB medium in the presence of 100 µg ml^−1^ ampicillin at 310 K to an OD_600_ of 0.5 (Table 1 ▸). The temperature was subsequently decreased to 293 K and protein overexpression was induced with 1.0 m*M* isopropyl β-d-1-thiogalactopyranoside (IPTG). After 18 h, the cells were harvested *via* centrifugation and resuspended in lysis buffer (50 m*M* NaH_2_PO_4_ pH 8.0, 300 m*M* NaCl, 20 m*M* imidazole, 1 m*M* lysozyme, 1 m*M* phenylmethanesulfonyl fluoride) at a ratio of 10 ml buffer per gram of pellet prior to lysis via sonication. After centrifugation at 95 000*g* and 277 K for 1 h, the supernatant was filtered through a 0.45 µm filter (Millipore) and subsequently loaded onto a 5 ml HisTrap FF column (GE Healthcare). UnGlnK was eluted with a linear gradient of His elution buffer (50 m*M* NaH_2_PO_4_ pH 8.0, 300 m*M* NaCl, 500 m*M* imidazole) over 15 column volumes (CV). Fractions containing the target protein were pooled and the His tag was cleaved off overnight at 289 K with thrombin at a ratio of 5 NIH units per milligram of unGlnK. Proteolytic cleavage was stopped by the addition of 1 m*M* 4-(2-aminoethyl)benzenesulfonyl fluoride. The sample was further purified via size-exclusion chromatography using a Superdex 75 16/600 column (GE Healthcare) pre-equilibrated in gel-filtration buffer (20 m*M* Tris–HCl pH 8.0, 200 m*M* NaCl, 1 m*M* EDTA). Fractions containing pure target protein, as verified by SDS–PAGE (Laemmli, 1970 ▸), were pooled, concentrated and used immediately in crystallization trials.

AdGlnK was expressed in *C. glutamicum* ATCC 13032 cells transformed via electroporation with the constitutive expression vector pZ8-1*glnK-Xa-C-Strep* (Dusch *et al.*, 1999 ▸; Table 1 ▸). Freshly transformed cells were grown on BHI agar in the presence of 15 µg ml^−1^ kanamycin at 303 K for 48 h. A single colony was used to inoculate a starter culture in BHI medium in the presence of 25 µg ml^−1^ kanamycin at 303 K for 6 h. Identical kanamycin concentrations and temperatures were used in all subsequent culturing steps. The starter culture was used to inoculate an overnight culture in kanamycin-containing CgC medium (Keilhauer *et al.*, 1993 ▸) in order to allow the adaptation of *C. glutamicum* to minimal medium. The main expression culture was inoculated at an OD_600_ of 1.0 in kanamycin-containing CgC medium and the cells were grown to an OD_600_ of 4.0–4.5. The cells were then washed twice *via* centrifugation followed by resuspension in CgCoN medium (Jakoby *et al.*, 2000 ▸) in order to remove any nitrogen sources and induce the adenylylation of GlnK. The cells were incubated for an additional 1.5 h in kanamycin-containing CgCoN before being harvested via centrifugation (5000*g*) at 277 K. The cells were resuspended in IEX buffer (20 m*M* Tris–HCl pH 6.5, 50 m*M* NaCl) supplemented with 10 mg ml^−1^ lysozyme and one cOmplete protease-inhibitor tablet (Roche) per 30 ml buffer at a ratio of 5 ml buffer per gram of pellet. The cell suspension was split into 1 ml aliquots in sterile 2 ml reaction tubes containing 300 mg of 0.2 µm glass beads and subjected to three consecutive 25 s cycles of high-frequency shaking at 6.5 m s^−1^ using a Precellys 24 tissue homogenizer (Bertin Technologies). The sample was chilled on ice for 2 min between cycles. The lysate was cleared by centrifugation at 95 000*g* and subsequent filtration through a 0.45 µm filter (Millipore). The cleared lysate was loaded onto a 1 ml SP Sepharose FF column (GE Healthcare) in order to remove lysozyme. The flowthrough fractions containing adGlnk were applied onto a 1 ml Q Sepharose FF column (GE Healthcare) and adGlnK was eluted via a gradient step of 33% IEX elution buffer (20 m*M* Tris–HCl pH 6.5, 1 *M* NaCl). Fractions containing the target protein were pooled, transferred into Mono Q buffer (20 m*M* Tris–HCl pH 7.0, 50 m*M* NaCl) using a desalting column and loaded onto a Mono Q 5/50 GL column (GE Healthcare). AdGlnK was eluted with a linear gradient of Mono Q elution buffer (20 m*M* Tris–HCl pH 7.0, 500 m*M* NaCl) over 30 CV and subjected to a final purification step using a Superdex 75 16/600 size-exclusion chromatography column pre-equilibrated with 20 m*M* Tris–HCl pH 7.5, 50 m*M* NaCl, 2 m*M* MgCl_2_. Fractions containing pure target protein, as verified by SDS–PAGE (Laemmli, 1970 ▸), were pooled, concentrated and used immediately in crystallization trials. Adenylylation was investigated and confirmed by mass spectrometry (Supplementary Fig. S1)

### 2.2. Crystallization   

UnGlnK was crystallized using the sitting-drop vapor-diffusion method by mixing 0.2 µl unGlnK (13 mg ml^−1^ unGlnK in 20 m*M* Tris–HCl pH 8.0, 200 m*M* NaCl, 1 m*M* EDTA) with 0.2 µl reservoir solution and equilibrating against 70 µl reservoir solution at 292 K (Table 2 ▸). Diffraction-quality crystals were obtained with a reservoir solution consisting of 2.0 *M* ammonium sulfate, 5%(*v*/*v*) 2-propanol.

Adenylylated GlnK was crystallized using the hanging-drop vapor-diffusion method by mixing 1 µl adGlnK (11 mg ml^−1^ adGlnK in 20 m*M* Tris–HCl pH 7.5, 50 m*M* NaCl, 2 m*M* MgCl_2_) with 1 µl reservoir solution and equilibrating against 700 µl reservoir solution at 292 K (Table 2 ▸). Diffraction-quality crystals were obtained with a reservoir solution consisting of 0.06 *M* MgCl_2_, 0.06 *M* CaCl_2_, 0.1 *M* 3-(*N*-morpholino)propanesulfonic acid (MOPS) pH 7.1, 0.1 *M* HEPES pH 7.1, 15%(*v*/*v*) 2-methyl-2,4-pentanediol (MPD), 15%(*w*/*v*) PEG 1000, 15%(*w*/*v*) PEG 3350.

### 2.3. Data collection and processing   

Crystals of unGlnK and adGlnK were flash-cooled in liquid nitrogen using 20%(*v*/*v*) ethylene glycol as a cryoprotectant. Diffraction data sets were collected from single crystals at 100 K on synchrotron beamline BL14.2 at BESSY II in Berlin to resolutions of 2.2 and 1.8 Å, respectively (Gerlach *et al.*, 2016 ▸). The data were indexed and integrated with *XDS* and scaled with *XSCALE* (Kabsch, 2010 ▸).

### 2.4. Structure determination   

Initial phases for the unGlnK and adGlnK data sets were obtained via molecular replacement with *Phaser* (McCoy *et al.*, 2007 ▸) using the structure of *Mycobacterium tuberculosis* nitrogen-regulatory P_II_ protein (PDB entry 3bzq; Shetty *et al.*, 2010 ▸) and the fully refined unGlnK structure as search models, respectively. The models were completed via alternating cycles of manual building in *Coot* and automated refinement with *Phenix* (Emsley *et al.*, 2010 ▸; Liebschner *et al.*, 2019 ▸). The quality of the final models was validated with *MolProbity* (Chen *et al.*, 2010 ▸).

### 2.5. Bioinformatics analyses   

Structure comparisons and superpositions were computed with either *LSQKAB* from the *CCP*4 suite or *DALI* (Winn *et al.*, 2011 ▸; Holm & Laakso, 2016 ▸). Solvent-accessible surface areas were calculated with *AREAIMOL*, and interaction energies were predicted with the *PISA* server (Winn *et al.*, 2011 ▸; Krissinel & Henrick, 2007 ▸). Sequence alignments were calculated with *PSI-BLAST* and analyzed using the *WebLogo* server (Crooks *et al.*, 2004 ▸; Camacho *et al.*, 2009 ▸). Interaction plots were generated with *LigPlot*+ (Laskowski & Swindells, 2011 ▸). All structure illustrations were produced with *UCSF Chimera* (Pettersen *et al.*, 2004 ▸).

## 3. Results   

### 3.1. Crystal structure of unGlnK   

**Figure 2:**
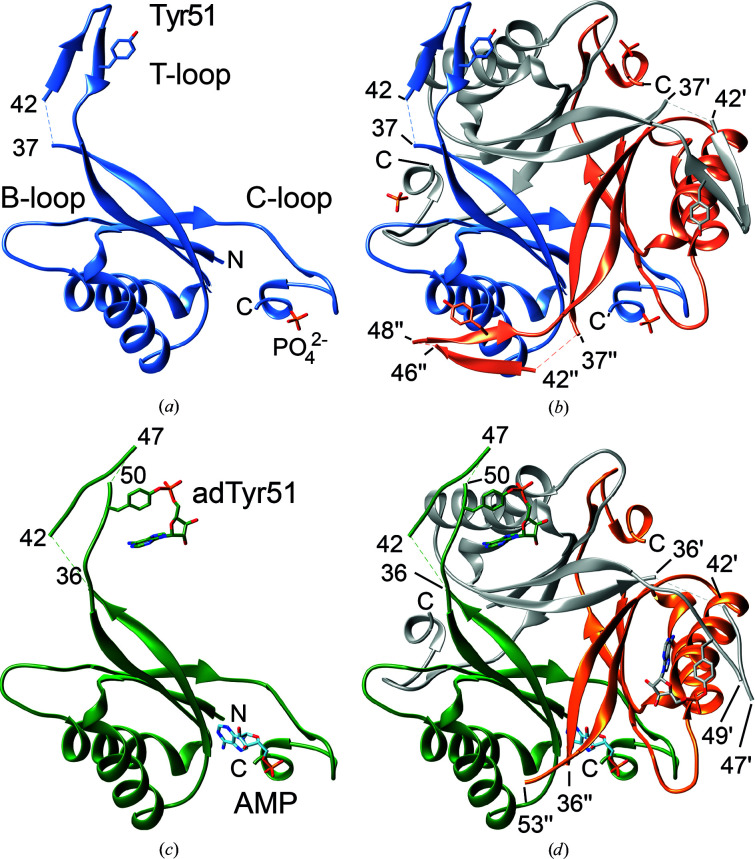
The three-dimensional structure of unGlnK and adGlnK from *C. glutamicum*. (*a*) Structure of an unGlnK monomer shown in a cartoon representation. The side chain of Tyr51, which is located in the T-loop (residues 37–55), as well as the phosphate ion bound in the ATP-binding pocket, is shown in a stick representation and labeled accordingly. (*b*) Structure of the unGlnK trimer. Primes and double primes denote residues from the second and third protomers, respectively. (*c*) Structure of one adGlnK monomer. The side chain of the adenylylated Tyr51, as well as the AMP bound in the ATP-binding pocket, is highlighted in a stick representation. (*d*) Structure of the adGlnK trimer.

The crystal structure of unGlnK from *C. glutamicum* was determined at 2.2 Å resolution (Fig. 2 ▸, Tables 3 ▸ and 4 ▸). The protein crystallized in the tetragonal space group *P*4_3_2_1_2 and the final model contains one GlnK trimer per asymmetric unit. Each GlnK subunit spans 112 residues and encompasses the three characteristic loop segments highlighted as important for function of the P_II_ protein (Forcada-Nadal *et al.*, 2018 ▸; Forchhammer & Selim, 2020 ▸). The T-loop (residues 37–55) extends from β-strands β2′ and β3′ and consists of an antiparallel β-finger structure formed by two short β-strands, named β2′′ (residues 42–46) and β3′′ (residues 49–53) that are interconnected by a β-turn with the sequence 46-YRGA-49 (Figs. 1 ▸ *a* and 2 ▸ *a*, Supplementary Fig. S2). Gly48 at position *i* + 2 of the β-turn appears to be strictly conserved among P_II_ proteins (Supplementary Figs. S2 and S3). The B-loop interconnects helix α2 and strand β4 and is formed by residues 82-RTG­KVGD-88 (Figs. 1 ▸ *a* and 2 ▸ *a*). The so-called C-terminal loop (C-loop; residues 102–106) extends from the end of β5 and includes part of β6. The latter strand is then followed by a single 3_10_-helical turn that ends with the C-terminus of the protein. All three chains present in the asymmetric unit could be traced continuously, with the exception of a four-amino-acid gap between residues 37 and 42 at the beginning of the T-loop, for which no electron density is observed in any of the three subunits of trimeric unGlnK (Fig. 2 ▸ *a*). Density is also lacking in one subunit for residue 47 located at the tip of the T-loop. The three subunits deviate from each other by an average r.m.s.d. value of 0.6 Å (C^α^ positions; Supplementary Table S1).

In the GlnK trimer, the secondary-structure elements of the subunits are extensively interlaced (Fig. 2 ▸ *b*). The surface area of isolated monomers amounts to 7550 Å^2^ on average and 2450 Å^2^ of this area becomes buried upon trimer formation (32% of the total solvent-accessible surface area of each monomer). The dissociation free energy is estimated by the *PISA* server to amount to 27 kcal mol^−1^ (Krissinel & Henrick, 2007 ▸). Hence, and as is the case for other P_II_ proteins, GlnK from *C. glutamicum* can be considered to be a permanent homotrimeric protein (Jones & Thornton, 1996 ▸; Nolden *et al.*, 2001 ▸). Inspection of the crystal packing suggests that trimeric GlnK further associates into hexamers, and identical hexamers can be observed in the crystal structures of both unGlnK and adGlnK (see below). The hexameric assembly is generated upon the application of a crystallographic twofold rotation along an axis that intersects the noncrystallographic threefold axis of the GlnK trimer at a right angle. Hence, the hexameric assembly displays *D*_3_ point-group symmetry (Fig. 1 ▸ *c*). In the hexamer, each monomer contributes an additional 850 Å^2^ of its surface to the oligomer interface. However, only trimers and not hexamers are observed in solution when purifying unGlnK and adGlnK via gel-filtration chromatography (data not shown).

Three phosphate molecules are bound to trimeric GlnK in unGlnK. Each phosphate binds to one of the three effector-binding sites located at the interfaces between the subunits (Figs. 1 ▸ *b* and 2 ▸). All three phosphates bind highly similarly and interact exclusively with residues from the B- and C-loops. The phosphates form a direct hydrogen bond to the backbone of Gly87 from the B-loop, as well as additional hydrogen bonds to the side chains of Arg101 and Arg103, which either precede or are part of the C-loop (Supplementary Figs. S2 and S4). In addition, water-bridged hydrogen bonds are formed to the backbone atoms of Arg103 and Gly89. These interactions closely resemble the interactions observed in P_II_ proteins when in complex with ATP and, more precisely, the inter­actions formed between the γ-phosphate group of ATP and the P_II_ protein, as for example observed in the ATP-bound structure of the P_II_ protein from *Aquifex aeolicus* (data not shown; PDB entry 2eg2; Rose *et al.*, 2017 ▸).

### 3.2. The GlnK fold and its quaternary structure are shared by many other P_II_ proteins   

GlnK from *C. glutamicum* displays high structural similarity to other P_II_ proteins. Searching the Protein Data Bank with *DALI* identifies about 80 related entries (Holm & Laakso, 2016 ▸). A nonredundant list of the ten closest homologues shows that GlnK from *C. glutamicum* shares the highest structural similarity with the P_II_ proteins from *M. tuberculosis*, *A. aeolicus*, *Azospirillum brasilense*, *Herbaspirillum seropedicae* and *Synechococcus elongatus*, with r.m.s.d. values ranging from 0.9 to 1.9 Å and sequence identities of between 45% and 65% (Supplementary Table S2). A multiple sequence alignment of these 11 structures reveals that, with one minor exception, all segments present in these proteins can be contiguously superimposed without the occurrence of insertions or deletions (Supplementary Fig. S2). The high degree of sequence conservation in P_II_ proteins in general is also apparent from a *WebLogo* representation of a multiple sequence alignment of as many as 197 different P_II_ proteins (Supplementary Fig. S3; Crooks *et al.*, 2004 ▸).

Inspection of the crystal packing of unGlnK and adGlnK revealed the existence of shared hexamers (Fig. 1 ▸ *c*). Of the ten closest structural homologues, the P_II_ protein GlnZ from *A. brasilense* (PDB entry 4co0; Rose *et al.*, 2017 ▸), the P_II_ protein from *Arabidopsis thaliana* (PDB entry 2o66; Mizuno *et al.*, 2007 ▸), GlnK1 from *Methanocaldococcus jannaschii* (PDB entry 2j9d; Yildiz *et al.*, 2007 ▸) and the P_II_ protein GlnK2 from *Archaeoglobus fulgidus* (PDB entry 3ncp; Helfmann *et al.*, 2010 ▸) also form hexameric assemblies (Supplementary Table S2). Hexamers can also be observed in P_II_ proteins extending beyond those identified as closest structural homologs, as is for example the case for the P_II_ protein from *Haloferax mediterranei* (PDB entry 4ozj; Palanca *et al.*, 2014 ▸). The proteins from *C. glutamicum*, *A. brasilense* (PDB entry 4co0) and *A. thaliana* (PDB entry 2o66) form identical hexamers, and hexamer formation is mediated by residues from the tip of the T-loop and via an antiparallel juxtaposition of two β3′ β-strands belonging to two different protomers and trimers (Fig. 1 ▸). Interestingly, in the hexameric assembly observed in GlnK1 from *M. jannaschii* (PDB entry 2j9d) and GlnK2 from *A. fulgidus* (PDB entry 3ncp), the same trimer interfaces are juxtaposed; however, the trimers are rotated with respect to each other when compared with the hexamer assembly observed in *C. glutamicum*. Moreover, in some crystals of ADP-bound GlnZ from *A. brasilense* (PDB entry 4cnz; Truan *et al.*, 2014 ▸), trimers of GlnZ assemble into hexamers via the opposite trimer interface (Truan *et al.*, 2014 ▸). While the biological function of the P_II_ trimers appears to be well established, the significance of these hexameric assemblies currently remains unclear.

### 3.3. Crystal structure of adGlnK   

The crystal structure of adGlnK was determined at 1.8 Å resolution (Fig. 2 ▸, Tables 3 ▸ and 4 ▸). The protein crystallized in the trigonal space group *P*3_2_ and the final model contains six proteins chains per asymmetric unit. The six adGlnK protomers form two homotrimers that assemble into a hexamer (Fig. 1 ▸ *c*). All six chains share highly similar overall conformations, and the average r.m.s. deviation obtained upon pairwise comparison of all six protomers is 0.33 Å (Supplementary Table S1).

**Figure 3:**
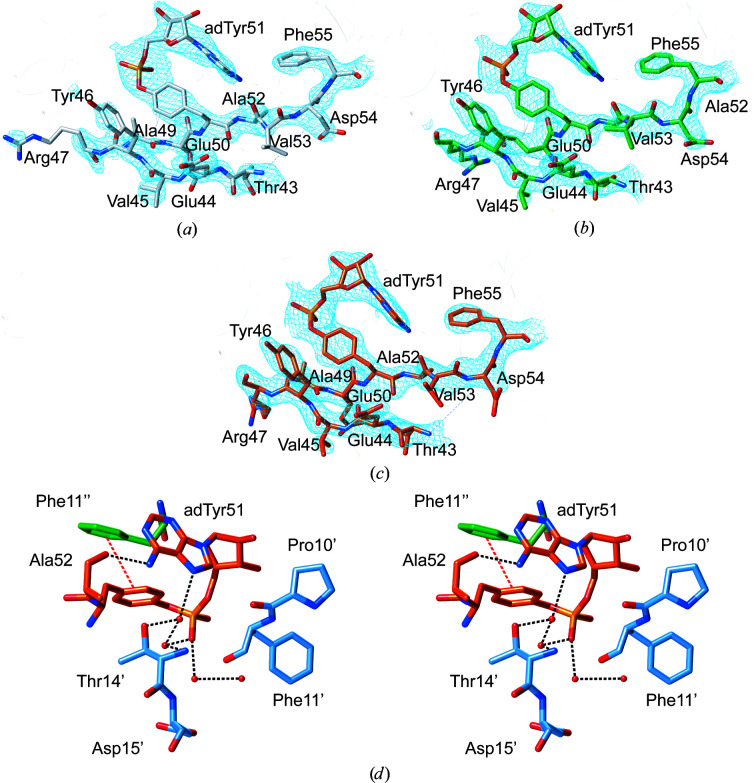
Detailed view of the adenylylated T-loop region (residues 43–55) in (*a*) chain *A* (with C atoms colored gray), (*b*) chain *B* (colored green) and (*c*) chain *E* (colored orange) of the adGlnK hexamer (chains *A*–*F*). The 2*mF*_o_ − *DF*_c_ electron-density map is shown in blue within a radius of 1.5 Å of any displayed atoms and is contoured at 1σ. (*d*) Stereo representation of the immediate surroundings of the adenylylated Tyr51 in chain *E*. Residues from chains *B*, *E* and *F* are colored green, blue and orange, respectively. Potential hydrogen bonds are shown as dotted black lines. The π–π-stacking interaction between the tyrosyl moiety of adTyr51 and Phe11′′ is shown as a dotted red line.

Electron density for the adenylylated Tyr51 residue can be observed in three of the six chains (Fig. 3 ▸). The AMP moiety, which is covalently linked to the hydroxyl group of Tyr51, curves back towards the protein backbone, where it is held in place via a hydrogen bond between the carbonyl group of the adjacent residue Ala52 and the terminal amino group of the adenine moiety (Fig. 3 ▸ *d*, Supplementary Figs. S2 and S5). Another stabilizing contact, which is conserved in all three modified tyrosines, is a π–π-stacking interaction between the aromatic rings of Tyr51 and Phe11 from an adjacent chain (Fig. 3 ▸ *d*). In addition, the phosphate moiety is tethered to the backbone via a network of water-bridged hydrogen bonds (Supplementary Fig. S5).

Mass-spectrometric measurements indicate that adGlnK is almost fully adenylylated (Supplementary Fig. S1). However, only three adenylyl moieties could be modeled with confidence in the adGlnK structure. The adenylylated Tyr51 residues are located very close to the twofold rotational symmetry axes that interrelate the trimers in the hexameric assembly (Fig. 1 ▸ *c*). If all six Tyr51 residues were modeled as fully adenylylated tyrosines then the pairs of AMP moieties would clash. Hence, only a single AMP moiety was modeled per two Tyr51 residues at each special position, namely the moiety that displayed the most easily interpretable electron density. This observation suggests that in the crystal, at any given point in time three adenylylated tyrosine residues are able to adopt defined conformations, while the other three residues are forced to adopt flexible conformations.

The crystals of adGlnK were assigned space group *P*3_2_. However, the data could alternatively also be reduced in space group *P*3_2_21, and a moderate decrease in *R*_p.i.m._ is observed when switching to the higher symmetry space group, namely 1.9% for *P*3_2_21 versus 2.5% for *P*3_2_ (Table 3 ▸ and data not shown). Molecular-replacement calculations yielded identical crystal-packing solutions in both space groups. However, whereas the asymmetric unit contains an entire adGlnK hexamer in space group *P*3_2_, the asymmetric unit is built up from trimers in space group *P*3_2_21. In the latter, the hexameric assembly is obtained upon the application of crystallographic twofold symmetry operations that are present in addition in space group *P*3_2_21. The diffraction data analysis of adGnK was performed in space group *P*3_2_ since more readily interpretable electron density was observed for the AMP moieties of the adenylylated Tyr51 residues and differences could also be observed with regard to the fortuitously bound effector molecules AMP and ADP in this space group (see below). At the same time, the crystallographic twofold symmetry axes in space group *P*3_2_21 introduced intermolecular clashes between the AMP moieties of the adenylylated Tyr51 residues (see above). Nonetheless, space group *P*3_2_21 cannot be fully ruled out as the correct space group at this time since a pairwise comparison of all six adGlnK monomers in *P*3_2_ shows that those monomer pairs that are related by dyads corresponding to crystallographic dyads in *P*3_2_21 and noncrystallographic dyads in *P*3_2_ display a lower r.m.s.d. value (0.12 Å on average) than those interrelated by the noncrystallographic threefold rotational symmetry axis that is present in both space groups (0.39 Å; Supplementary Table S1).

### 3.4. Fortuitous effector binding in adGlnK   

The hexamer that is present in the asymmetric unit of the adGlnK crystals encompasses six effector-binding sites. While four of these sites appear to be devoid of any ligand, fortuitous binding of AMP and ADP was observed in the remaining two binding sites (Supplementary Fig. S6). In both the AMP- and ADP-binding sites, the adenine nucleobase is bound *via* two hydrogen bonds to the backbone of Ile64 from strand β3 and via one hydrogen bond to the side chain of Thr29 displayed from strand β2. Thr29 further forms a hydrogen bond to the hydroxyl group attached to atom C2′ of the ribose moiety of AMP or ADP (Supplementary Figs. S6 and S7). This interaction pattern between the nucleoside moiety and the P_II_ protein is highly conserved among AMP-, ADP- and ATP-bound P_II_ proteins and can, for example, also be found in the AMP-bound structure of SbtB from *Cyanobacterium* sp. 7001 (PDB entry 6mmo; Kaczmarski *et al.*, 2019 ▸) and the ADP-bound structure of GlnK2 from *H. mediterranei* (PDB entry 4ozj; Palanca *et al.*, 2014 ▸), as well as in the ATP-bound structure of the P_II_ protein from *A. aeolicus* (PDB entry 2eg2; Rose *et al.*, 2017 ▸). Moreover, Thr29 is among the most highly conserved residues in P_II_ proteins (Supplementary Fig. S3).

In the AMP-binding site of adGlnK, the α-phosphate group forms two direct hydrogen bonds to the backbone N atoms of Gly87 and Gly89 displayed from the B-loop and in addition an indirect and water-bridged hydrogen-bond interaction with the side chain of Arg101 from the C-loop (Supplementary Figs. S6*a* and S7*a*). With some variations, this α-phosphate-binding mode is also highly conserved among P_II_ proteins. However, in some P_II_ proteins, such as for example SbtB from *Cyanobacterium* sp. 7001 (PDB entry 6mmo), the α-phosphate group interacts with side chains from residues displayed from strand β4 rather than from the preceding B-loop (data not shown).

In the ADP-bound effector-binding site the α-phosphate group is slightly displaced, and of the two glycines Gly87 and Gly89, the α-phosphate group forms only a single hydrogen bond to Gly87, while the additional β-phosphate moiety interacts via a water-bridged hydrogen bond with the side chain of Lys58 from β-strand β3 located at the end of the T-loop (Supplementary Figs. S6*b* and S7*b*). While this kind of interaction mirrors that in the binding of ADP to GlnK2 from *H. mediterranei* (PDB entry 4ozj; Palanca *et al.*, 2014 ▸), in other ADP-bound P_II_ proteins the terminal phosphate is often bound directly by one to three serine residues displayed from strand β4 and, more importantly, by residues from the T-loop (data not shown). Moreover, in the latter proteins the tight interactions between ADP and the T-loop appear to stabilize a specific T-loop conformation that is characterized by a Gln39–Lys58 side-chain interaction (Truan *et al.*, 2010 ▸). This conformation is often referred to as the canonical ADP-bound T-loop conformation and is distinct from that observed in the absence of bound nucleotides (Truan *et al.*, 2014 ▸). In the case of adGlnK, however, residue Gln39 cannot be modeled and no differences in the conformation of the T-loop can be observed, irrespective of whether the effector-binding site is empty or is occupied by AMP or ADP (see below).

Of the six T-loops present in the six P_II_ monomers in the asymmetric unit of the adGlnK crystals, a considerably higher number of T-loop residues could be modeled in those loops in which the adenylylated Tyr51 residues are clearly resolved, and a considerably lower number of T-loop residues are visible in the two monomers where the effector-binding site is occupied by either AMP or ADP (Supplementary Fig. S8). This observation suggests that nucleotide binding possibly increases the conformational flexibility of the adenylylated T-loop and is in line with a similar observation for uridylylated GlnB from *E. coli* (Palanca & Rubio, 2017 ▸). In uridylylated GlnB from *E. coli*, the effector-binding site is occupied by ATP, and similarly to the binding of AMP and ADP to GlnK, only the base of the T-loop is ordered (Supplementary Fig. S8*c*). At the same time, in both adenylylated GlnK and uridylylated GlnB the nucleotides bind to the respective P_II_ protein in a highly similar fashion (Supplementary Fig. S8).

### 3.5. Structural comparison between unGlnK and adGlnk   

The overall fold of the monomers and the assembly of trimers and hexamers are virtually identical in unGlnK and adGlnK. A cross-comparison of the monomers from the unGlnK and adGlnK crystals shows that the r.m.s.d. values (0.49 Å on average) are almost identical to those obtained when superimposing unGlnK or adGlnK monomers separately (0.6 and 0.33 Å, respectively, see above; Supplementary Table S1).

**Figure 4:**
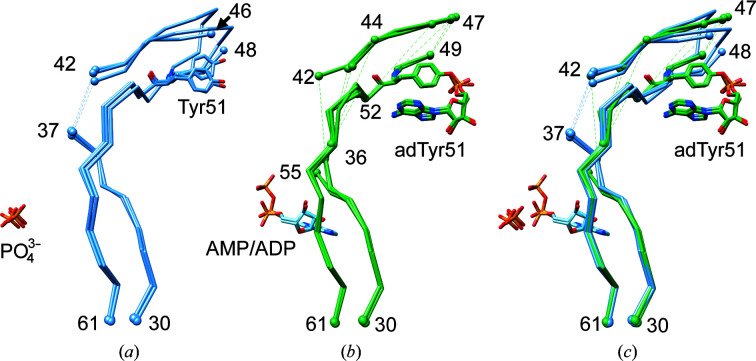
T-loop conformations in unGlnK and adGlnK. Superposition of the T-loops from (*a*) all three monomers in the unGlnK trimer, (*b*) all six monomers present in adGlnK and (*c*) all monomers from both unGlnK and adGlnK. In all panels the T-loops were superimposed using the coordinates of the entire monomers.

Such a high degree of similarity is to be expected for the core regions of the GlnK trimers, given the level of structural conservation among P_II_ proteins. However, a close inspection of the T-loops also reveals a highly similar overall positioning of the T-loop in all nine independently observed monomers, namely in the three monomers in the unGlnK crystal structure and the six monomers in adGlnK (Fig. 4 ▸). In addition, the presence or absence of ligands, whether it be phosphate ions, AMP or ADP, appears to have little effect on the conformation of the T-loop in the present structures. This also holds true for the adenylylation of Tyr51, which also does not appear to affect the conformation of the T-loop. The only sizeable difference between the different monomers is that in the presence of a bound AMP or ADP molecule a lower number of T-loop residues can be modeled in the respective protomers (Fig. 4 ▸ and Supplementary Fig. S8).

## 4. Discussion   

In this work, a protocol for the production of adenylylated adGlnK has been established. While the present study focuses on the determination of the crystal structures of adGlnK and unGlnK, the high yields obtained for adGlnK will greatly facilitate future additional in-depth characterizations of adGlnK. The post-translationally adenylylated Tyr51 residue is well resolved in the crystal structure of adGlnK, and the atomic interactions formed between the AMP moiety and the T-loop can be observed in great detail. So far, this has not been possible for any other post-translationally modified P_II_ protein. For example, in the crystal structure of uridylylated GlnB from *E. coli*, local disorder prevented the modeling of the uridylylated tyrosine residue (Palanca & Rubio, 2017 ▸).

In addition to the post-translational modification, three fortuitously bound ligands were observed in the effector-binding sites of both unGlnK and adGlnK. In unGlnK all three effector-binding pockets were occupied by single phosphate molecules, and in adGlnK two of the six binding pockets were occupied by either AMP or ADP despite the fact that no nucleotides were added to the crystallization buffer. It is possible that the presence of AMP and ADP in adGlnK hints at differences in the effector-binding affinities between adGlnK and unGlnK. However, such an interpretation must be made with caution since the two proteins were produced in different bacterial expression systems using different media and purification protocols. Interestingly, in the crystal structure of uridylylated GlnB from *E. coli*, ATP was bound in the effector-binding site. In contrast to adGlnK, however, ATP was deliberately added to the crystallization solution of uridylylated GlnB (Palanca & Rubio, 2017 ▸). The question as to whether adenylylation alters the nucleotide-binding affinity of GlnK (or likewise whether uridylylation alters the affinity of GlnB) is certainly of importance when aiming at better understanding the function of GlnK (and GlnB), but would require the determination of the exact effector-binding affinities. These experiments could also help to clarify whether AMP is a genuine effector of GlnK from *C. glutamicum*.

Depending on the nature of the bound ligands, the T-loop adopts distinct and specific conformations in P_II_ proteins (Truan *et al.*, 2014 ▸). Of particular interest are the extended conformation that is adopted upon binding ADP and the compacted conformation that is induced upon the binding of Mg-ATP and 2OG. While the ADP-bound conformation promotes the binding of P_II_ proteins to interaction partners, the formation of the complex with Mg-ATP and 2OG abrogates binding in general (Fokina *et al.*, 2010 ▸; Truan *et al.*, 2010 ▸; Forchhammer & Selim, 2020 ▸). The so-called canonical ADP-bound conformation is observed in the crystal structures of complexes formed between the P_II_ protein GlnZ and DraG, GlnK and AmtB and in the P_II_–PipX complex, while in the only other additionally structurally characterized complex, namely that of P_II_ in complex with NagK, ATP alone was present in the effector-binding site of the P_II_ protein (Forcada-Nadal *et al.*, 2018 ▸; Forchhammer & Selim, 2020 ▸).

**Figure 5:**
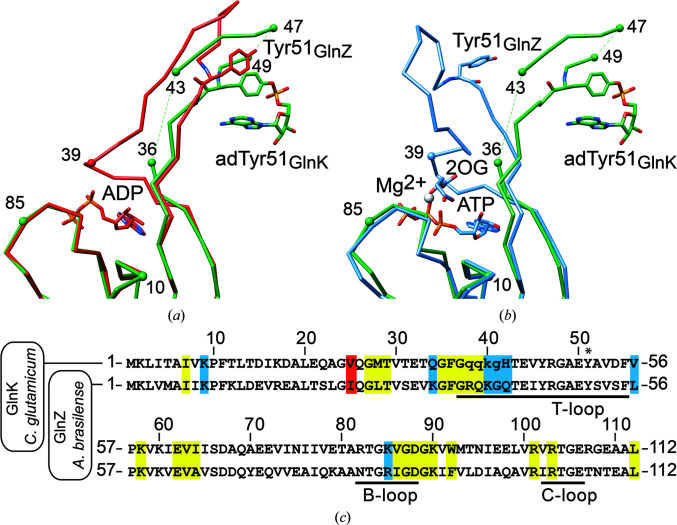
Comparison of effector binding and T-loop conformation between adGlnK from *C. glutamicum* and GlnZ from *A. brasilense*. (*a*) Superposition of adGlnK (in green) and ADP-bound GlnZ (red; PDB entry 4co1; Truan *et al.*, 2014 ▸). (*b*) Superposition of adGlnK (in green) and Mg-ATP and 2OG-bound GlnZ (blue; PDB entry 3mhy; Truan *et al.*, 2010 ▸). For clarity, only a single subunit is shown and hence the contribution of the B- and C-loop residues to effector binding is not shown. (*c*) Sequence alignment between GlnK and GlnZ. Residues located within 4.5 Å of any bound effector atom are highlighted in red, blue and yellow when involved solely in ADP binding, solely in Mg-ATP/2OG binding or in binding both ADP and Mg-ATP/2OG, respectively

The T-loop conformations observed in unGlnK and adGlnK resemble the canonical ADP-bound conformation, albeit with some differences (Figs. 4 ▸ and 5 ▸). Whereas residues 38–41, which are located at the base of the T-loop, are disordered in unGlnK and adGlnK, these residues are well ordered in the canonical ADP-bound conformation and directly participate in ADP binding, as for example observed in the ADP-bound complex of GlnZ from *A. brasil­ense* (Fig. 5 ▸; Truan *et al.*, 2014 ▸). A direct comparison of the residues located within 4.5 Å of any ADP atom in GlnZ with the corresponding residues in GlnK from *C. glutamicum* suggests that GlnK should be able to adopt the canonical ADP-bound conformation, since sequence differences mainly occur in residues that contribute to ADP binding in *A. brasilense* via main-chain interactions (data not shown) (Fig. 5 ▸ *c*). Moreover, adenylyl­ation of Tyr51 also does not appear to interfere with the conformation of the T-loop; hence, adGlnK and unGlnK should be equally capable of adopting the canonical ADP-bound conformation (Fig. 5 ▸ *a*).

Of similar importance for P_II_ function is the compacted T-loop conformation observed in P_II_ proteins bound to Mg-ATP and 2OG (Truan *et al.*, 2010 ▸; Fig. 5 ▸ *b*). All attempts to crystallize a ternary complex consisting of Mg-ATP, 2OG and either unGlnK or adGlnK, however, have so far failed. Considerations similar to those discussed above for the canonical ADP-bound conformation strongly suggest that GlnK from *C. glutamicum* is likely to bind Mg-ATP and 2OG, and moreover is also able to adopt a similar compacted T-loop conformation. The sequence identity is very high between the residues directly involved in effector binding, as exemplified by comparing the Mg-ATP and 2OG complex of GlnZ from *A. brasilense* with GlnK from *C. glutamicum* (Fig. 5 ▸ *c*; Truan *et al.*, 2010 ▸). The most pronounced sequence difference is the substitution of Arg38 in GlnZ by glutamine in GlnK. In GlnZ, the main-chain NH group of Arg38 directly interacts with the β-phosphate of ATP, while its guanidino group interacts with the γ-phosphate. From this, it appears likely that Gln38 in GlnK can take over the role of Arg38 in GlnZ in ATP binding. Interestingly, adenylylation of Tyr51 is also not expected to interfere with the T-loop conformation in the case of the compacted conformation, since Tyr51 remains fully accessible at the protein surface (Fig. 5 ▸ *b*).

In all presently structurally characterized P_II_-protein complexes, post-translational modification of the P_II_ protein either abrogated binding to a partner protein, as seen for example for the interaction of GlnK with AmtB in *E. coli* upon the uridylylation of GlnK and for the interaction of *S. elongatus* P_II_ with NAGK upon the phosphorylation of *S. elongatus* P_II_, or had no effect on the interaction, as for example proposed for the P_II_–PipX interaction upon the phosphorylation of P_II_ residue Ser49 (Merrick, 2014 ▸). In the case of GlnK from *C. glutamicum*, it has been proposed that adenylylation of Tyr51 is a prerequisite for the interaction of GlnK with its binding partner, namely the bacterial repressor protein AmtR (Beckers *et al.*, 2005 ▸). AmtR functions as a global repressor and regulates the transcription of multiple genes involved in nitrogen metabolization (Jakoby *et al.*, 2000 ▸). In this context, adGlnK has been proposed to function as an inducer of AmtR and to alleviate gene repression by abrogating DNA operator binding of AmtR (Beckers *et al.*, 2005 ▸). Based on the crystal structure of *C. glutamicum* AmtR, a model for interaction of AmtR with adGlnK has been proposed. In this model, two adGlnK trimers bind to an AmtR hexamer and block the binding of the AmtR operator (Sevvana *et al.*, 2017 ▸).

A similar model has also been proposed for the interaction of *E. coli* GlnB with glutamine synthetase adenylyl transferease (ATase; Palanca & Rubio, 2017 ▸). In the case of GlnB, uridylylation of Tyr51 is a prerequisite for complex formation. Interestingly, in both adenylylated GlnK and uridylylated GlnB, post-translational modification of the P_II_ protein does not engender defined conformational rearrangements in the respective P_II_ protein, hinting that complex formation is not modulated by an allosteric mechanism but that rather direct atomic interactions between the adenylylated Tyr51 of adGlnK and AmtR as well as between the uridylylated Tyr51 of GlnB and ATase promote complex formation (Palanca & Rubio, 2017 ▸).

So far, however, it has not been possible to reconstitute an adGlnk–AmtR complex *in vitro*, irrespective of whether or not additional effector molecules were present. Neither the addition of millimolar concentrations of ADP, ATP alone or ATP together with 2OG resulted in the formation of an adGlnK–AmtR complex that could be monitored by gel-filtration chromatography or observed in pull-down assays. Clearly, further experiments are needed to further understand the function of adGlnK.

## Supplementary Material

PDB reference: GlnK, adenylylated, 6yc6

PDB reference: unadenylylated, 6yc7

Supplwmentary Tables and Figures. DOI: 10.1107/S2059798321000735/jv5002sup1.pdf

## Figures and Tables

**Table 1:** Macromolecule-production information

	UnGlnK	AdGlnK
Source organism	*C. glutamicum*	*C. glutamicum*
DNA source	*C. glutamicum* genomic DNA	*C. glutamicum* genomic DNA
Forward primer	GCCAATTGTACCATATGAGCTTGCATGCCTGC†	CCATGCGGATTAAAGGGCTGCTTCGCCGC‡
Reverse primer	GGGCCGCTCGAGTCATCAAAGGGCTGCTTCGCC§	GCGGCGAAGCAGCCCTTTAATCCGCATGG‡
Cloning vector	pZ8-1*glnK-Xa-C-Strep*	pZ8-1*glnK-Xa-C-Strep*
Expression vector	pET-15b*glnK*	pZ8-1*glnK*
Expression host	*E. coli* BL21(DE3)	*C. glutamicum* ATCC 13032
Complete amino-acid sequence of the construct produced	MGSSHHHHHHSSGLVPRGSHMSLHACRMKLITAIVKPFTLTDIKDALEQAGVQGMTVTETQGFGQQKGHTEVYRGAEYAVDFVPKVKIEVIISDAQAEEVINIIVETARTGKVGDGKVWMTNIEELVRVRTGERGEAAL	MKLITAIVKPFTLTDIKDALEQAGVQGMTVTETQGFGQQKGHTEVYRGAEYAVDFVPKVKIEVIISDAQAEEVINIIVETARTGKVGDGKVWMTNIEELVRVRTGERGEAAL

† The restriction site for NdeI is underlined.

‡ The inserted stop codon is underlined.

§ The restriction site for XhoI is underlined.

**Table 2:** Crystallization

	UnGlnK	AdGlnK
Method	Sitting-drop vapor diffusion	Hanging-drop vapor diffusion
Plate type	Greiner CrystalQuick 96-well	ComboPlate 24-well
Temperature (K)	292	292
Protein concentration (mg ml^−1^)	13	11
Buffer composition of protein solution	20 m*M* Tris–HCl pH 8.0, 200 m*M* NaCl, 1 m*M* EDTA	20 m*M* Tris–HCl pH 7.5, 50 m*M* NaCl, 2 m*M* MgCl_2_
Composition of reservoir solution	2.0 *M* ammonium sulfate, 5%(*v*/*v*) 2-propanol	0.06 *M* MgCl_2_, 0.06 *M* CaCl_2_, 0.1 *M* MOPS pH 7.1, 0.1 *M* HEPES pH 7.1, 15%(*v*/*v*) MPD, 15%(*w*/*v*) PEG 1000, 15%(*w*/*v*) PEG 3350
Volume and ratio of drop	0.4 µl, 1:1	2.0 µl, 1:1
Volume of reservoir (µl)	70	700

**Table 3:** Data collection and processing Values in parentheses are for the outer shell.

	UnGlnK	AdGlnK
Diffraction source	BL14.2, BESSY II	BL14.2, BESSY II
Wavelength (Å)	0.9184	0.9184
Temperature (K)	100	100
Space group	*P*4_3_2_1_2	*P*3_2_
*a*, *b*, *c* (Å)	82.51, 82.51, 170.59	53.44, 54.44, 179.95
α, β, γ (°)	90, 90, 90	90, 90, 120
Mosaicity (°)	0.11	0.41
Resolution range (Å)	48.16–2.20 (2.28–2.20)	22.97–1.80 (1.86–1.80)
Total No. of reflections	265594 (24581)	271662 (27397)
No. of unique reflections	30720 (2999)	53142 (5326)
Completeness (%)	99.9 (100.0)	99.6 (99.8)
Multiplicity	8.6 (8.2)	5.1 (5.1)
〈*I*/σ(*I*)〉	15.3 (1.2)	17.5 (1.4)
*R*_p.i.m._	0.036 (0.645)	0.025 (0.527)
Overall *B* factor from Wilson plot (Å^2^)	48.3	30.0

**Table 4:** Structure solution and refinement Values in parentheses are for the outer shell.

	UnGlnK	AdGlnK
PDB code	6cy6	6cy7
Resolution range (Å)	48.16–2.20 (2.28–2.20)	22.41–1.80 (1.86–1.80)
Completeness (%)	99.9 (100.0)	99.6 (99.8)
No. of reflections, working set	30714 (2999)	53100 (5325)
No. of reflections, test set	1540 (148)	1288 (123)
Final *R*_cryst_	0.208 (0.308)	0.187 (0.278)
Final *R*_free_	0.234 (0.326)	0.223 (0.308)
No. of non-H atoms		
Protein	2480	4687
Ligand	47	93
Water	54	278
Total	2581	5086
R.m.s. deviations		
Bond lengths (Å)	0.004	0.009
Angles (°)	0.57	0.96
Average *B* factors (Å^2^)		
Overall	59.0	43.2
Protein	58.9	43.7
Ligand	72.7	70.8
Water	51.0	42.4
Ramachandran plot		
Most favored (%)	98.7	99.5
Allowed (%)	1.3	0.5
