# Supplementary material for: Crystal structures of adenylylated and unadenylylated PII protein GlnK from Corynebacterium glutamicum
Source: Acta Crystallogr D Struct Biol. 2021 Feb 19;77(Pt 3):325–35. doi: 10.1107/S2059798321000735 (PMC7919409; doi:10.1107/S2059798321000735)
Supplement: Supplementary file 1 [file d-77-00325-sup1.pdf]

STRUCTURAL
BIOLOGY

Volume 77 (2021)

Supporting information for article:

Crystal structures of adenylylated and unadenylylated PII protein GlnK from *Corynebacterium glutamicum*

Florian C. Grau, Andreas Burkovski and Yves A. Muller

Table S1

R.m.s.d. values of the structural comparison of all unGlnK and adGlnK monomers present in the crystallographic asymmetric units of the two crystal structures.

	unGlnK monomers (chain IDs)				adGlnK monomers (chain IDs)					
	A	B	C		A	B	C	D	E	F
unGlnK										
A	-	0.70[a]	0.35		0.51	0.60	0.57	0.41	0.63	0.53
B	108	-	0.75		0.45	0.44	0.42	0.40	0.43	0.41
C	107	107	-		0.50	0.58	0.49	0.42	0.64	0.46
adGlnK										
A	105	105	104		-	0.42	0.41	0.14	0.39	0.39
B	105	105	104		104	-	0.36	0.38	0.13	0.35
C	96	96	96		96	96	-	0.42	0.36	0.10
D	100	100	100		100	100	96	-	0.39	0.41
E	105	105	104		105	104	96	100	-	0.36
F	94	94	94		94	94	94	94	94	-

[a] R.m.s.d. values (in Å) are reported above the diagonal and the number of structurally aligned Cα-positions below the diagonal. R.m.s.d. values were calculated based on Cα-positions with program LSQKAB from the CCP4 program package (Winn *et al.*, 2011).

Table S2

Non-redundant Z-score-ranked list of unGlnK-homologous structures present in the protein databank as identified using the Dali webserver.[a]

Dali rank[b]	PDB-entry[c]	UNIPROT entry[d]	Z-score	r.m.s.d. [Å]	no. of align. resid.	seq. identity [%][e]	protein length [no. of residues][f]	protein identity
1	3bzq	P9WN31	18.1	0.9	95	65	99	P_{II} protein from *Mycobacterium tuberculosis*
2	2eg2	O66513	17.8	0.9	95	58	95	P_{II} protein from *Aquifex aeolicus*
3	4co0	P70731	17.8	1.0	107	53	109	P_{II} protein GlnZ from *Azospirillum brasilense*
4	1hwu	P94852	17.3	1.9	99	48	99	P_{II} protein from *Herbaspirillum seropedicae*
5	4c3k	P0A3F4	17.3	0.9	95	47	98	P_{II} protein from *Synechococcus elongatus*
6	1gnk	P0AC55	17.3	1.3	98	55	98	P_{II} protein GlnK from *Escherichia coli*
7	2j9d	Q60381	17.2	1.3	98	50	101	P_{II} protein GlnK1 from *Methanocaldococcus jannaschii*
8	2o66	Q9ZST4	17.0	0.8	96	45	106	P_{II} protein from *Arabidopsis thaliana*
9	3ncp	O28527	16.6	1.7	99	47	102	P_{II} protein GlnK2 from *Archaeoglobus fulgidus*
10	5l9n	P0A9Z1	16.6	0.9	92	49	92	Uridylylated P_{II} protein GlnB from *Escherichia coli*

[a] Refs: (Holm & Laakso, 2016; Rose *et al.*, 2017)
[b] The coordinates of chain A of unGlnK were used as search coordinates for identifying the closest structural homologues of unGlnK. The list was arbitrarily truncated after 10 entries.
[c] In case multiple entries of the same protein, i.e. different effector-bound complexes, were identified by the Dali search then only the entry with the highest Z-score was retained. This also applies to the presence of multiple copies of identical protein chains in the same entry.
[d] Ref: (The UniProt Consortium, 2017)
[e] Sequence identities in the protein segments used in the structure-based alignment.
[f] Total number of residues present in the deposited protein model.

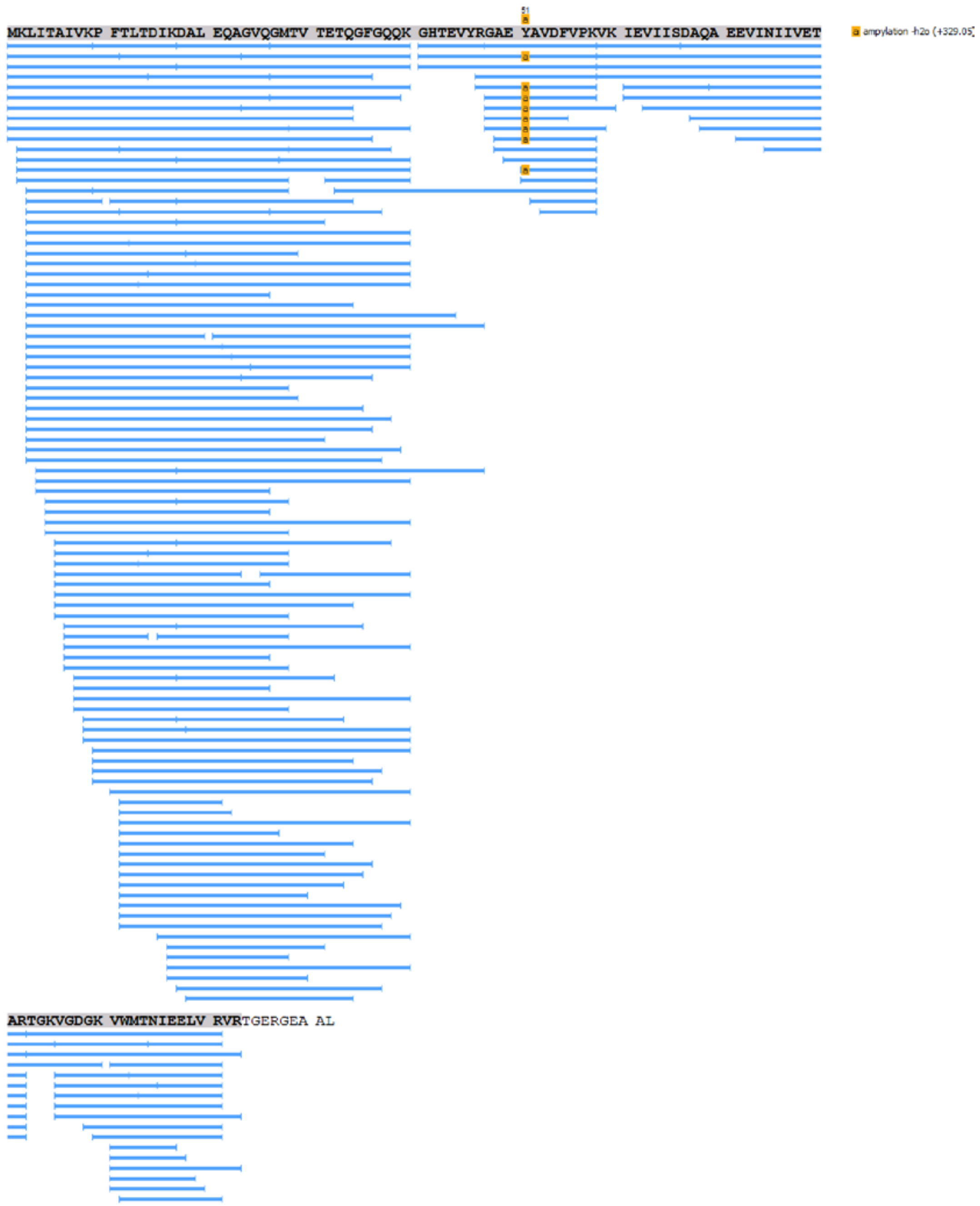

Figure S1

Peptide coverage of adGlnK as determined by mass spectrometry with the adenylylation shown in yellow. The depiction of peptides is not quantitative. The adenylylation rate was estimated to be close to 99%.

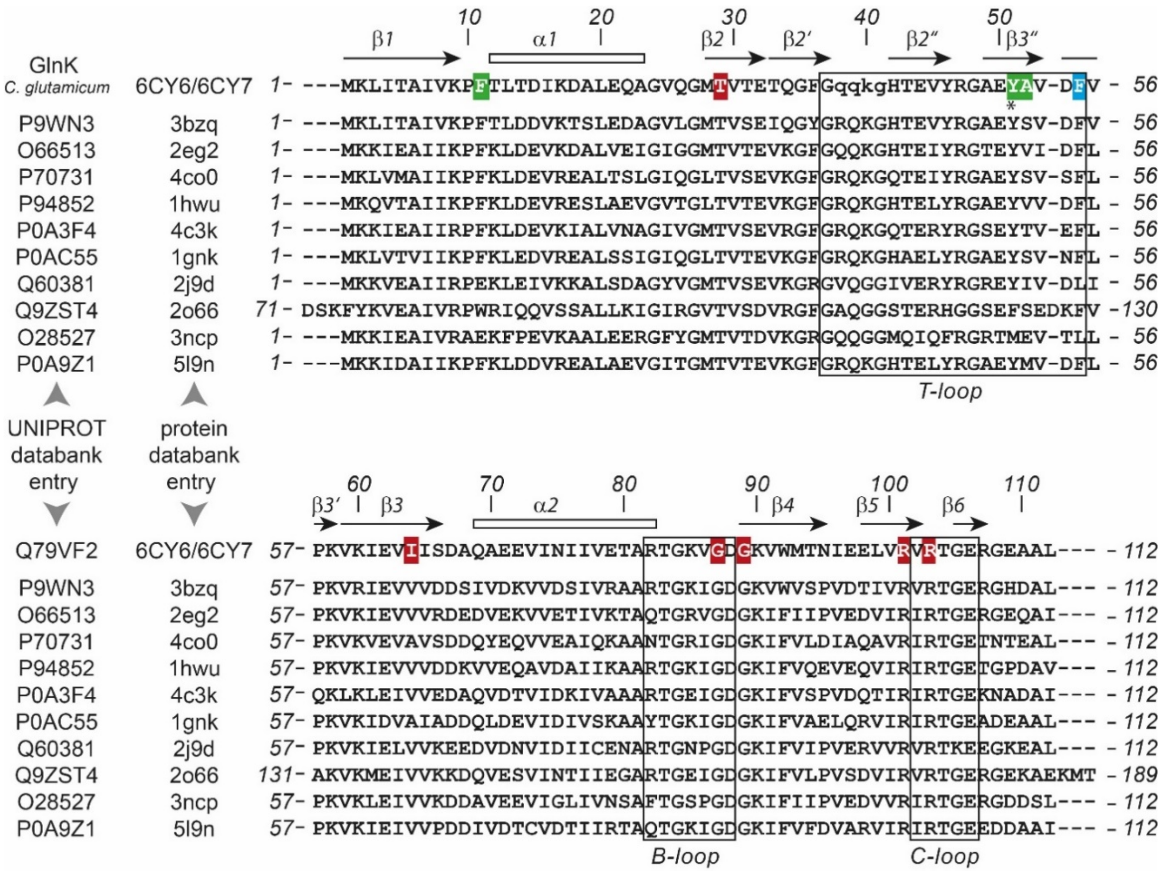

Figure S2

Structure-based annotated sequence alignment of GlnK from *C. glutanicum* and of the ten closest structurally homologous P_{II} proteins as identified with program DALI (see also Supplementary Table S2). Residues explicitly named in the main text are colored as follows: in green, residues interacting with the adenylylated Tyr51 residue; in blue, a residue involved in stabilizing the hexameric assembly *via* inter-subunit main chain hydrogen bonds and in red, residues involved in phosphate, AMP or ADP binding. Residue numbering for protein data bank entry 2o66 corresponds to that of UNIPROT entry Q9ZST4. It differs form that used in the deposited coordinate file for entry 2o66.

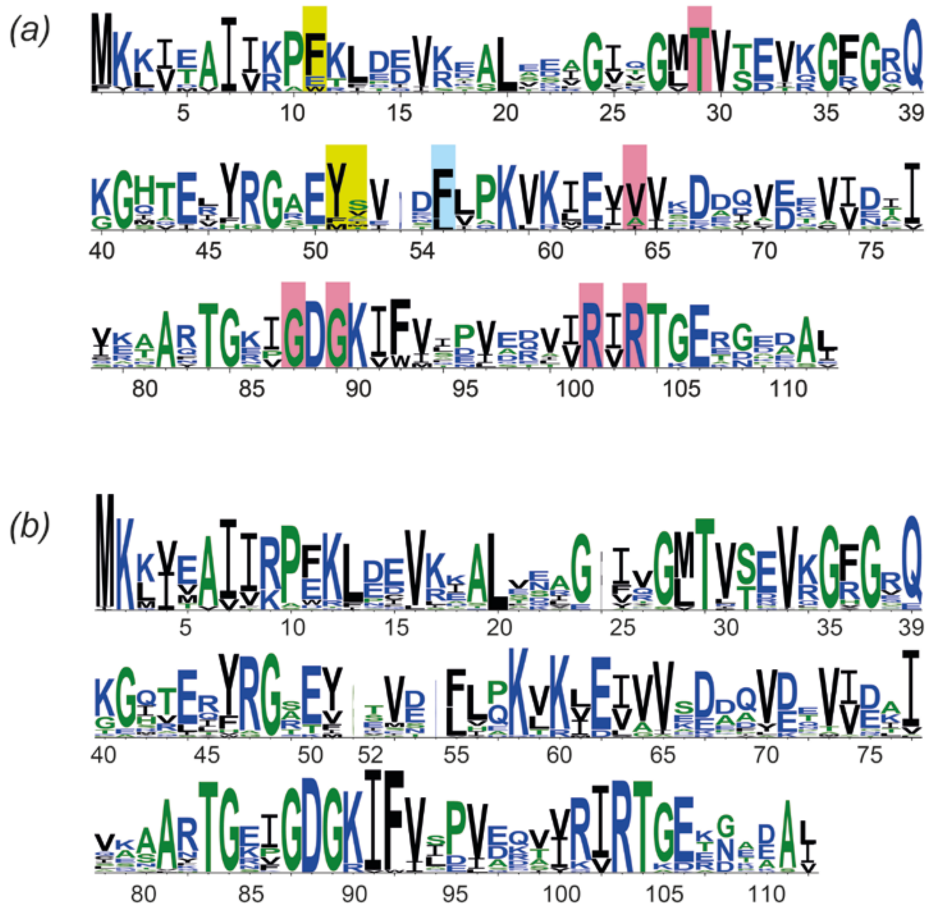

Figure S3

Sequence conservation analyses as performed with program WebLogo 3 (http://weblogo.threeplusone.com/create.cgi; (Crooks *et al.*, 2004)) (*a*) Weblogo analysis of the alignment of the eleven sequences displayed in Supplementary Fig. S2. Selected residues are highlighted using a very similar color coding as in Supplementary Fig. S2 (*b*) Weblogo analysis/representation of a multiple sequence alignment of 197 P_{II} proteins identified with ProtBLAST/PSI-BLAST (Camacho *et al.*, 2009). The degree of conservation is reflected in character size. In both panels, residue numbering adheres to the amino acid sequence numbering of GlnK from *C. glutamicum*.

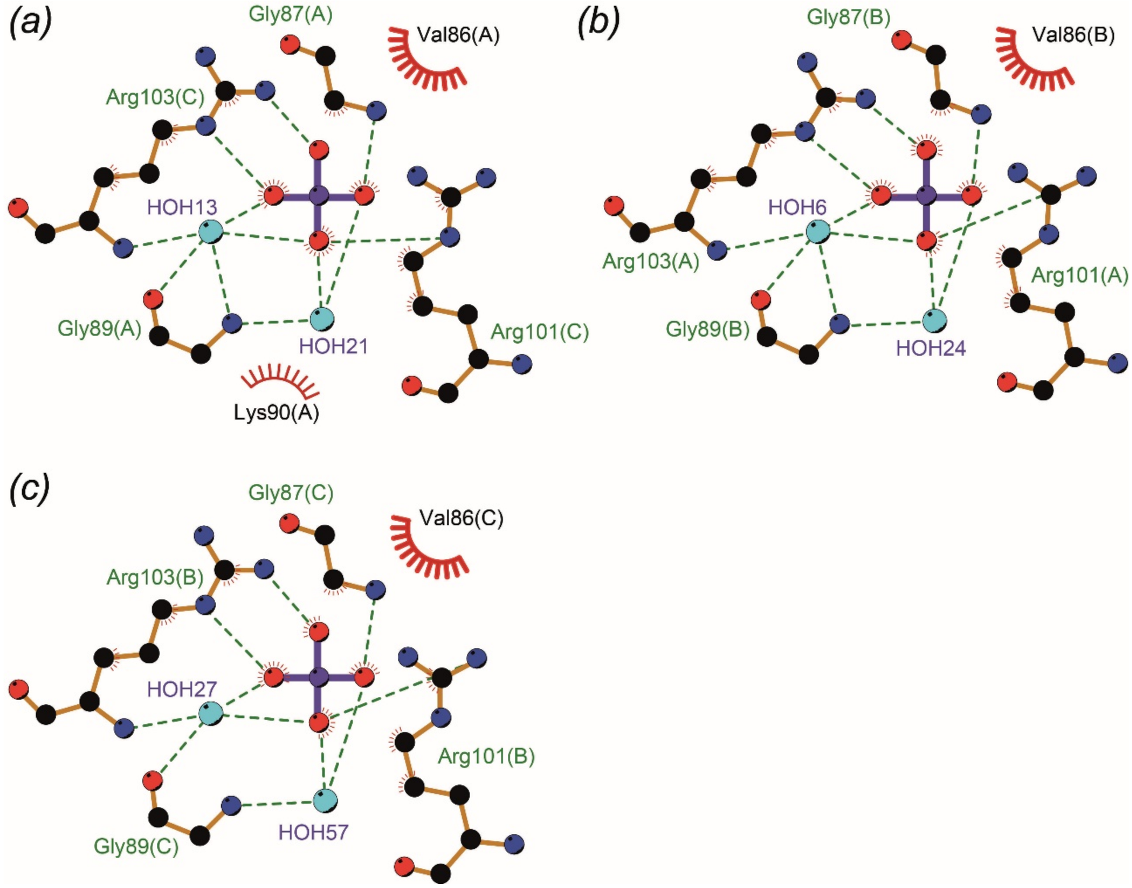

Figure S4

Interaction plots of the phosphate ions in the ATP-binding pockets towards the C-termini of chains (*a*) A and C, (*b*) A and B and (*c*) B and C generated with program Ligplot+ (Laskowski & Swindells, 2011). Hydrogen bonds are shown as dotted green lines. Residues, which participate in hydrophobic interactions, are highlighted by red circle segments.

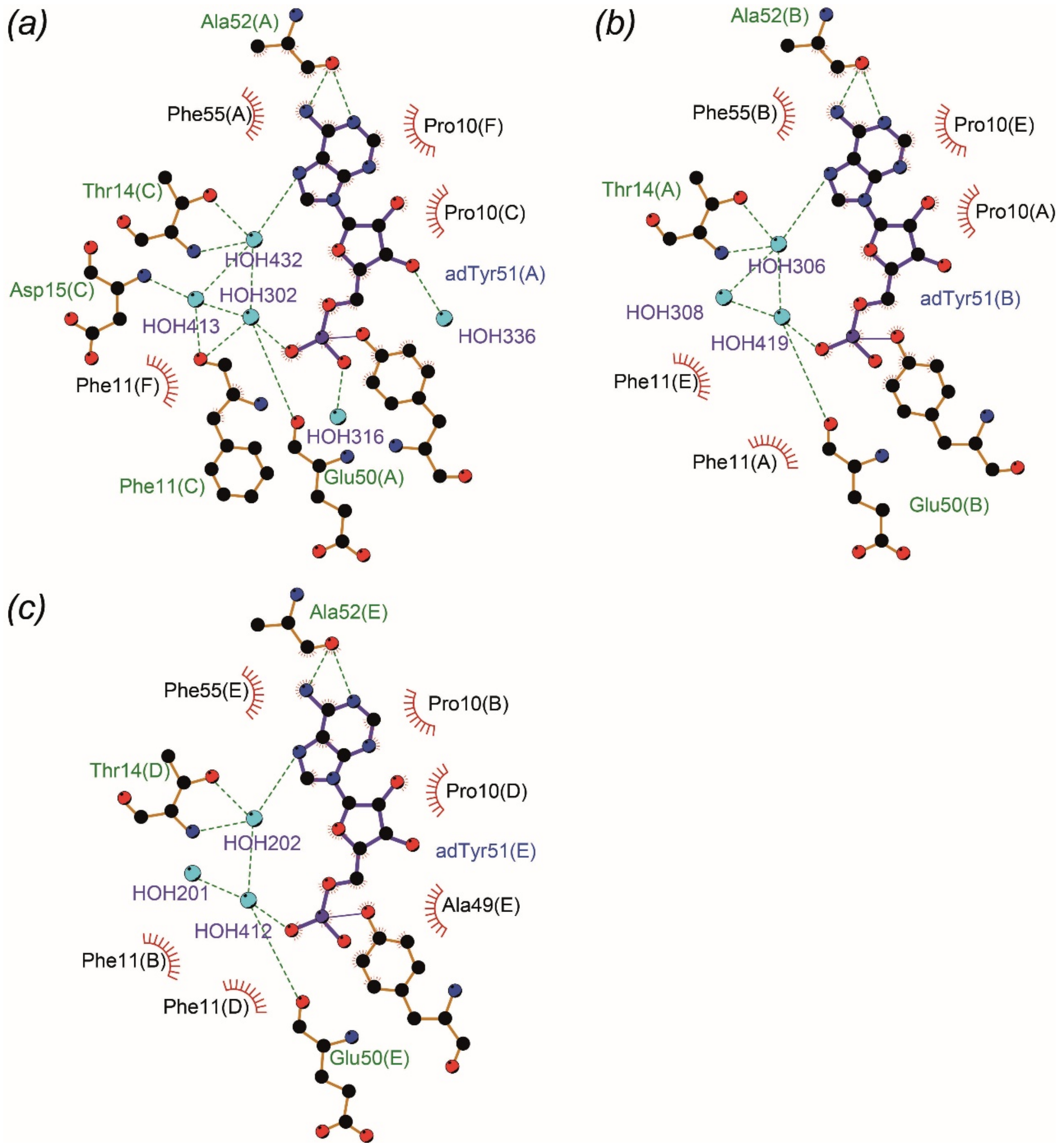

Figure S5

Interaction plots of the adenylylated tyrosine residues in (*a*) chain A, (*b*) chain B and (*c*) chain E generated with program Ligplot+ (Laskowski & Swindells, 2011). Covalent bonds are shown as solid purple lines, and hydrogen bonds are shown as dotted green lines. Residues, which participate in hydrophobic interactions with the ligand, are indicated by red circle segments.

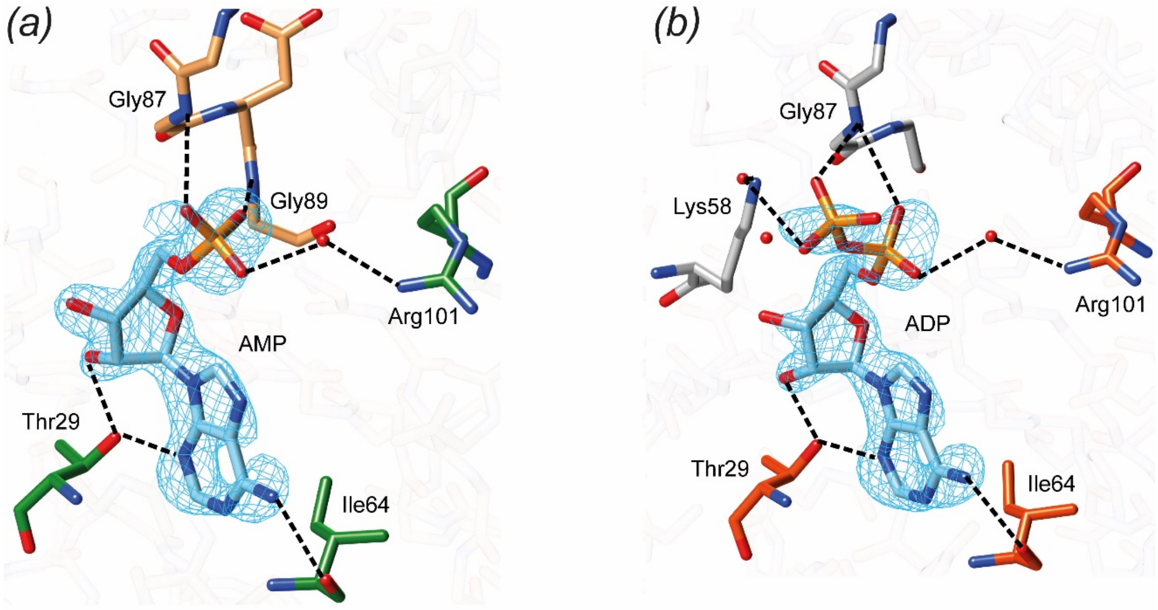

Figure S6

Detailed view of (*a*) the ATP-binding pocket located between chains B (colored green) and C (colored beige) of one adGlnK trimer with the bound AMP molecule shown in blue and (*b*) the ATP-binding pocket located between chains F (colored orange) and G (colored gray) of the second adGlnK trimer with the bound ADP molecule shown in blue. Amino acid residues interacting directly with the ligand are shown in stick representations. The $2mF_O$-DF_C electron density map is shown in blue within a radius of 1.5 Å of any ligand atom and is contoured at 1σ. Electron density for the surrounding residues was omitted for clarity. Hydrogen bonds are shown as dotted black lines.

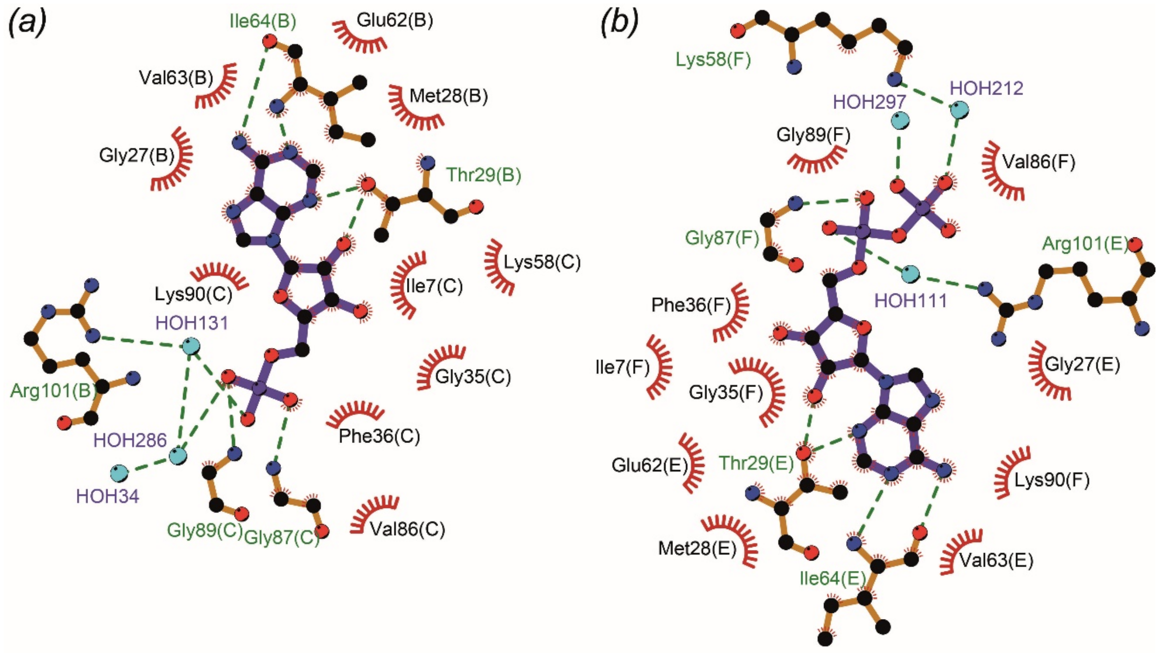

Figure S7

Interaction plots of (*a*) AMP and (*b*) ADP bound to adGlnK generated with program Ligplot+ (Laskowski & Swindells, 2011). Hydrogen bonds are shown as dotted green lines. Residues, which participate in hydrophobic interactions with the ligand, are highlighted by red circle segments.

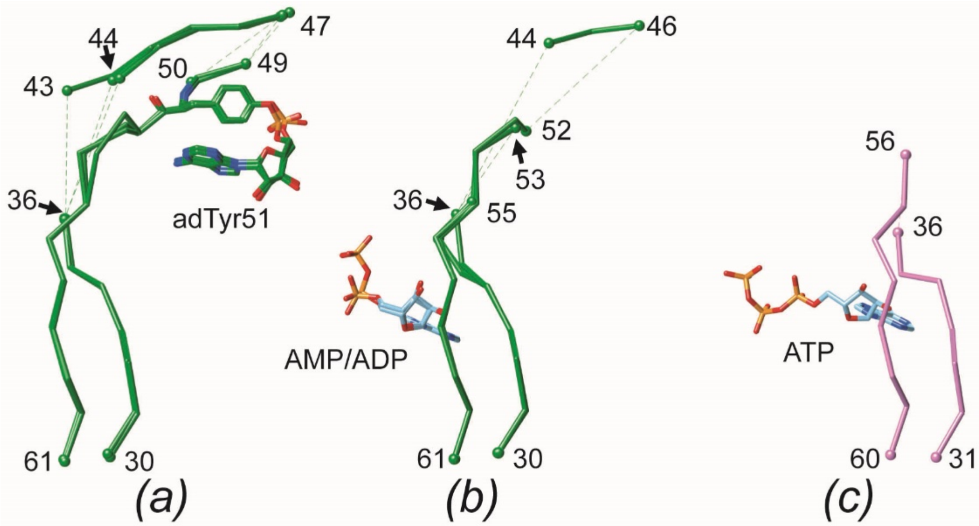

Figure S8

Comparison of the T-loop conformations in adenylylated GlnK and in uridylylated GlnB. *(a)* T-loop conformation in those three monomers present in the asymmetric unit of adGlnK in which adenylylated Tyr51 could be modelled. *(b)* T-loop conformation in those three monomers in adGlnK in which the adenylylated Tyr51 could not be modelled. In two of these three monomers, fortuitously bound nucleotides, i.e. either AMP or ADP, could be observed. *(c)* T-loop conformation in uridylylated GlnB with a bound ATP molecule (PDB code 5l9n, (Palanca & Rubio, 2017)).

<u>Supplementary references</u>

Camacho, C., Coulouris, G., Avagyan, V., Ma, N., Papadopoulos, J., Bealer, K. & Madden, T. L. (2009). *BMC Bioinformatics* **10**, 421.

Crooks, G. E., Hon, G., Chandonia, J. M. & Brenner, S. E. (2004). *Genome Res* **14**, 1188-1190.

Holm, L. & Laakso, L. M. (2016). *Nucleic Acids Res* **44**, W351-355.

Laskowski, R. A. & Swindells, M. B. (2011). *J Chem Inf Model* **51**, 2778-2786.

Palanca, C. & Rubio, V. (2017). *Environ Microbiol Rep* **9**, 290-299.

Rose, P. W., Prlic, A., Altunkaya, A., Bi, C., Bradley, A. R., Christie, C. H., Costanzo, L. D., Duarte, J. M., Dutta, S., Feng, Z., Green, R. K., Goodsell, D. S., Hudson, B., Kalro, T., Lowe, R., Peisach, E., Randle, C., Rose, A. S., Shao, C., Tao, Y. P., Valasatava, Y., Voigt, M., Westbrook, J. D., Woo, J., Yang, H., Young, J. Y., Zardecki, C., Berman, H. M. & Burley, S. K. (2017). *Nucleic Acids Res* **45**, D271-D281.

The UniProt Consortium (2017). *Nucleic Acids Res* **45**, D158-D169.

Winn, M. D., Ballard, C. C., Cowtan, K. D., Dodson, E. J., Emsley, P., Evans, P. R., Keegan, R. M., Krissinel, E. B., Leslie, A. G., McCoy, A., McNicholas, S. J., Murshudov, G. N., Pannu, N. S., Potterton, E. A., Powell, H. R., Read, R. J., Vagin, A. & Wilson, K. S. (2011). *Acta Crystallogr D Biol Crystallogr* **67**, 235-242.
